# A matter of delicate balance: Loss and gain of Cockayne syndrome proteins in premature aging and cancer

**DOI:** 10.3389/fragi.2022.960662

**Published:** 2022-07-21

**Authors:** Elena Paccosi, Adayabalam S. Balajee, Luca Proietti-De-Santis

**Affiliations:** ^1^ Unit of Molecular Genetics of Aging, Department of Ecology and Biology, University of Tuscia, Viterbo, Italy; ^2^ Cytogenetic Biodosimetry Laboratory, Radiation Emergency Assistance Center/Training Site, Oak Ridge Institute of Science and Education, Oak Ridge Associated Universities, Oak Ridge, TN, United States

**Keywords:** Cockayne Syndrome, aging, cancer, transcription coupled repair, premature aging syndromes

## Abstract

DNA repair genes are critical for preserving genomic stability and it is well established that mutations in DNA repair genes give rise to progeroid diseases due to perturbations in different DNA metabolic activities. Cockayne Syndrome (CS) is an autosomal recessive inheritance caused by inactivating mutations in CSA and CSB genes. This review will primarily focus on the two Cockayne Syndrome proteins, CSA and CSB, primarily known to be involved in Transcription Coupled Repair (TCR). Curiously, dysregulated expression of CS proteins has been shown to exhibit differential health outcomes: lack of CS proteins due to gene mutations invariably leads to complex premature aging phenotypes, while excess of CS proteins is associated with carcinogenesis. Thus it appears that CS genes act as a double-edged sword whose loss or gain of expression leads to premature aging and cancer. Future mechanistic studies on cell and animal models of CS can lead to potential biological targets for interventions in both aging and cancer development processes. Some of these exciting possibilities will be discussed in this review in light of the current literature.

## Introduction

Aging is a complex biological process characterized by the gradual reduction of tissue, organ and cellular homeostasis. In this context, senescent cells, by virtue of their altered chromatin structure coupled with gene expression profiles, have been suggested to play a pivotal role in mediating impaired tissue regeneration, organismal aging and age-associated diseases. On the other hand, somatic and stem cells may acquire advantageous mutations over time that may facilitate the development of certain oncogenic properties such as sustained proliferative signaling and resistance to cell death, thus eventually leading to the onset of cancer. While genomic instability is known to be a convergent driving force for both aging and cancer, it is still unclear what factors determine the cell fate between tumorigenesis and senescence.

DNA repair genes are the guardians of genomic stability and their functions decline during the normal aging process. The molecular link between DNA repair deficiency and aging is strongly supported by some of the human premature aging syndromes resulting from mutations in well-known DNA repair genes. These progeroid syndromes serve as an ideal model system for understanding the role of DNA repair in aging process since most symptoms observed during normal aging process in healthy humans are similar to humans afflicted with premature aging diseases (Kamenisch and Berneburg, 2009). Moreover, in most cases, patients afflicted with premature aging syndromes display also an early and increased cancer incidence, illustrating that genomic instability, driven by DNA repair deficiency, can promote both aging and carcinogenic processes (Schumacher et al., 2008; Wolters and Schumacher, 2013). Mutations in three of the five human RecQ helicases (WRN, BLM and RecQL4) are yet known to result in premature aging syndromes characterized by increased cancer predisposition: Werner, Bloom and Rothmund-Thomson. Cells derived from these patients have been found to be defective in various DNA repair pathways (see Balajee, 2021 and references therein). Interestingly, a fourth member of the RecQ family of helicases, RecQL1, has been recently associated with a human genome instability disorder, named RECON (RECqlONe) syndrome. RECON patients display progeroid facial features, xeroderma, and photosensitivity (Abu-Libdeh et al., 2022).

Additionally, there are two classical Nucleotide Excision Repair (NER) deficient syndromes, Xeroderma Pigmentosum (XP) and Cockayne Syndrome (CS), which can be included in the growing list of premature aging syndromes. There are eight complementation groups for XP (A, B, C, D, E, F, G, and Variant) and two complementation groups for CS (A and B). All the proteins of XP and CS play crucial roles in NER pathway. Although XP and CS patients are extremely sensitive to ultraviolet radiation due to NER deficiency, increased cancer incidence is only seen in XP patients.

### Nucleotide excision repair deficient syndromes

**FIGURE 1:**
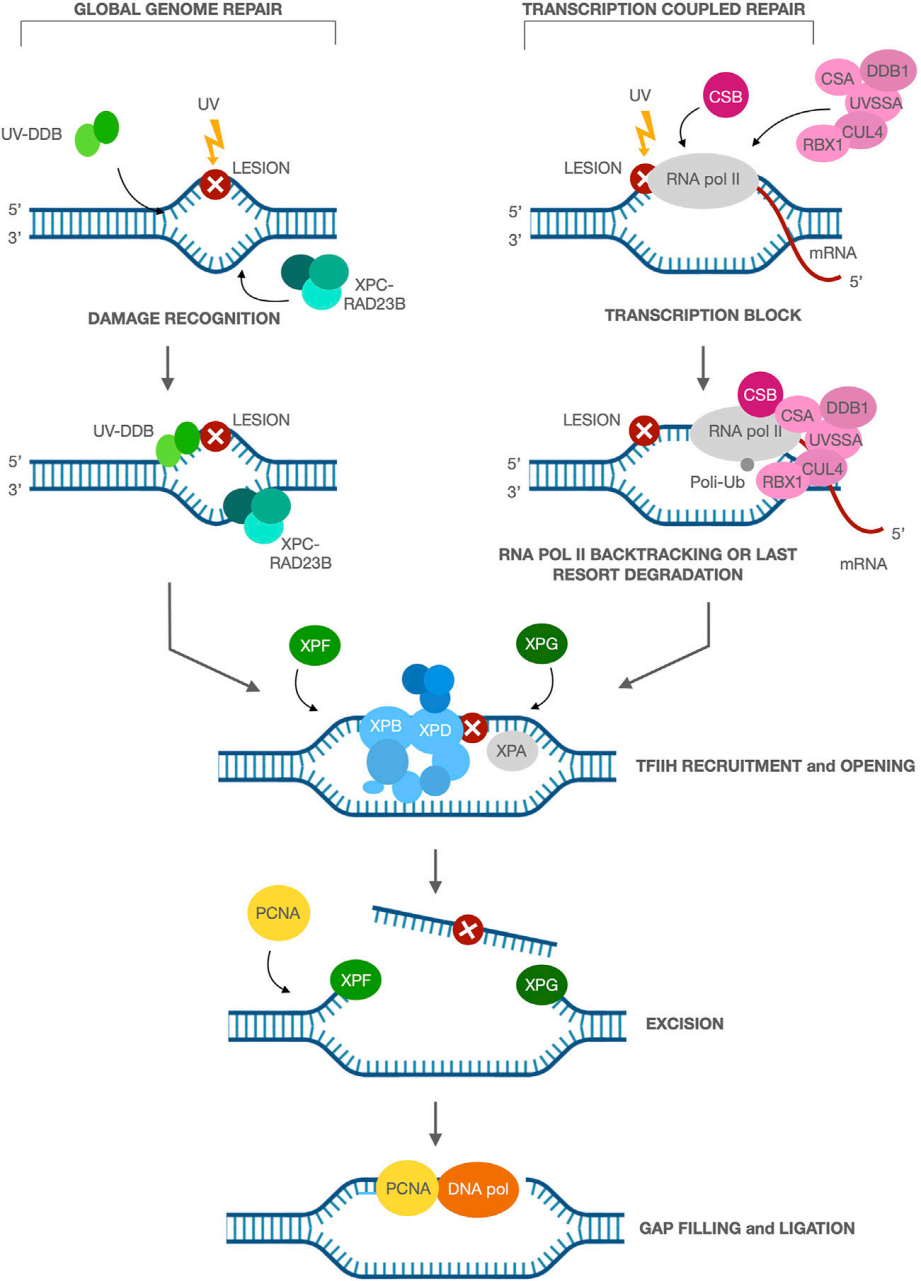
Nucleotide excision repair and its two sub-pathways. Global genome repair (GGR): the UV-induced DNA damage is recognized and removed by GGR, that involves several proteins acting in tandem, including the UV-DDB and XPC-RAD23B, involved in damage recognition. Transcription coupled repair (TCR): TCR is triggered when elongating RNA pol II is blocked by DNA damage in the transcribed strand. CSB is involved in the initiation of TCR through recognition of blocked RNA pol II and binding to this complex, followed by recruitment of the other NER proteins CSA, CUL4, RBX1, DDB1 and UVSSA to the damaged site. In both the sub-pathways, XPB and XPD helicases, which are part of TFIIH, are recruited at the lesion for the DNA unwinding. XPA protein maintains the open DNA region containing the damage, which is then cut out by XPF/ERCC1 and XPG endonucleases at the 3′ and 5′ ends of the damaged DNA, respectively. PCNA recruitment next favors the DNA polymerase action in filling in the resulting gap.

Nucleotide Excision Repair (NER) is a major pathway for the removal of bulky DNA adducts such as those induced by UV. NER pathway consists of different steps: recognition of damaged DNA, incision/excision of damaged DNA, resynthesis of new DNA substituting the damaged DNA and ligation. NER operates at two levels: Global genome repair (GGR) and transcription coupled repair (TCR) with different kinetics (Hanawalt and Spivak, 2008). TCR facilitates the rapid repair of DNA lesions induced on the transcribing strand of active genes to promote cell survival by resumption of transcription. The UV-induced DNA photoproducts cyclobutane pyrimidine dimers (CPD) and 6-pyrimidine-4-pyrimidone products are recognized and removed by GGR that involves several proteins acting in tandem, including double-strand DNA–binding protein 2 (DDB2) and XPC. After the recognition, the DNA is unwound by XPB and XPD helicases, which are part of the 10-subunit basal transcription factor IIH (TFIIH). The XPA protein maintains the open DNA region containing the damage, which is then cut out by XPF/ERCC1 and XPG endonucleases at the 3′ and 5’ ends of the damaged DNA, respectively. The resulting gap is filled in by DNA polymerase and ligase. TCR is triggered when elongating RNA polymerase II (RNA pol II) is blocked by DNA damage in the transcribed strand. RNA pol II complex must be displaced and/or degraded for an efficient repair to occur because blocked polymerase II complex can shield and prevent the accessibility of NER proteins to the lesion sites (Licht et al., 2003; Fousteri and Mullenders, 2008). Interestingly, a recent and elegant work of Kokic and colleagues, based on both previously published data and their new Cryo-electron microscopy, suggests that the ATPase activity of Cockayne Syndrome group B protein (CSB) is able to push the backtrack of RNA pol II forward enabling it to resume elongation if the block can be bypassed. If the block cannot be bypassed, CSB is then responsible for the recruitment of Cockayne Syndrome group A (CSA) and its associated DNA damage binding protein 1 (DDB1), Cullin 4A and Roc 1 (Rbx1) E3 ubiquitin ligase complex (CRL4^CSA^) to the site of damage-stalled RNA pol II (Lainé and Egly, 2006; Vermeulen and Fousteri, 2013). The recruitment of UV Stimulated Scaffold Protein A (UVSSA) to stalled Pol II depends on CSA (van der Weegen et al., 2020). CRL4^CSA^ ubiquitylates RNA pol II at K1268, leading to the recruitment of TFIIH near UVSSA and enabling DNA repair with the same cascade of GGR (Figure 1). Finally, rearrangement of CRL4^CSA^ leads to the polyubiquitylation of CSB and degradation by the proteasome (Anindya et al., 2010), which releases TCR factors that are all anchored via CSB, enabling RNA Pol II to resume transcription. Alternatively, the persistence of stalled RNA pol II may trigger a last resort mechanism, in which RNA pol II is ubiquitinated and degraded in a CSA and CSB dependent manner (Wilson et al., 2013; Kokic et al., 2021).

The NER pathway requires coordinated activities of multiple proteins and this is the reason why mutations in any of NER genes either the TCR or GGR sub-pathways lead to abnormalities in DNA repair. Besides DNA repair deficiency, mutations in NER genes lead to multiple clinical syndromes with overlapping features, including Xeroderma Pigmentosum (XP), Cockayne Syndrome (CS), Cerebro-Oculo-Facial-Skeletal syndrome (COFS), and Trichothiodystrophy (TTD) (Black, 2016).

### Xeroderma pigmentosum

XP is associated with mutations in one of the eight complementation groups: XPA, XPB/ERCC3, XPC, XPD/ERCC2, XPE/DDB2, XPF/ERCC4, XPG/ERCC5, and XPV/POLH. Xeroderma Pigmentosum (XP) is an autosomal recessive disorder and the XP patients are highly photosensitive since XP gene products play crucial roles in NER pathway (van Steeg and Kraemer, 1999; DiGiovanna and Kraemer, 2012). The prevalence of XP is variable, affecting 1 per million in the United States, 2.3 per million in Western Europe, and 45 per million in Japan. The shorter average lifespan of XP patients depends both on skin cancer and neurodegeneration, but the minimization of UV radiation exposure can improve the course of disease and prolong life (Black, 2016).

XP patients are not protected from UV light at an early age and therefore develop photodamage and vision impairment including blindness besides an elevated risk for different types of skin cancers (Bradford et al., 2011). UV radiation damage leads to an early onset and increased frequency of both nonmelanoma skin cancer (NMSC) and melanoma in XP patients, with a few differences between the types. Indeed, XPC, XPE and XPV types have been associated with less severe Sun burning after minimal Sun exposure, but still acquire abnormal pigmentation (Bradford et al., 2011; Tamura et al., 2014). The anatomic distribution of NMSC in XP patients is similar to that in the general population, with over 80% occurring on the face, head, and neck (Kraemer et al., 1994). XP patients display a drastic and at early age increase in cancers of the brain and other organs of the central nervous system (Kraemer et al., 1994), including brain medulloblastoma (Giannelli et al., 1981), glioblastoma, spinal cord astrocytoma (DiGiovanna et al., 1998), and Schwannoma. The spectrum of this disease also includes a severe form characterized by dwarfism, gonadal hypoplasia, and mental deficiency along with the conventional features of XP (de Sanctis and Cacchione, 1932) and neurologic abnormalities (Neisser, 1883), which are second only to cancer for causing the death of XP patients.

### Cockayne syndrome

Cockayne syndrome (CS) is a rare autosomal recessive disorder linked to mutations in the *ERCC8* and *ERCC6* genes encoding for Cockayne syndrome protein A (CSA) and Cockayne syndrome protein B (CSB), respectively (Troelstra et al., 1992; Henning et al., 1995) both of which play a role in TCR (Bregman et al., 1996; Svejstrup, 2003). CS is characterized by progressive neurodegeneration, mental retardation, developmental abnormalities, retinal degeneration, physical impairment, severe photosensitivity and premature aging (Karikkineth et al., 2017). The phenotype of the patients is subdivided into three types based on the severity of symptoms: i) the type I corresponds to the moderate phenotype, in which life expectancy is 16 years; ii) the type II is the most severe and with the earliest onset, with a life expectancy of 5 years; iii) the type III is the form with the highest life expectancy (above 30 years) and in which the phenotype manifests itself later in life (Laugel, 2000).

CSB protein exhibits ATPase activity and CSB belongs to SWI2/SNF2 family of chromatin remodelers (Citterio et al., 2000; Batenburg et al., 2017). Recently, it was shown that CSB possesses an ubiquitin binding domain (Anindya et al., 2010). CSA belongs to the family of WD-40 repeat proteins, known for coordinating the interactions in multiprotein complexes (Zhang and Zhang, 2015) and is a component of the ubiquitin E3 ligase complex, containing CUL4, RBX1 and DDB1 (Groisman et al., 2003; Fischer et al., 2011).

In sharp contrast to XP patients, cancer incidence has not been reported in CS patients despite a demonstrated deficiency in TCR. While mutational loss of functions lead to a different spectrum of abnormalities, mostly correlated with stress-induced cell death and/or cell senescence, increased expression of CS proteins has been reported in cancer cells from different tissues often associated with increased proliferation and cell robustness due to the induction of pro survival pathways (Spyropoulou et al., 2021). In this context, it has been recently demonstrated that inhibition of CS proteins is sufficient to halt neoplastic growth (Caputo et al., 2013; Paccosi et al., 2021; Filippi et al., 2022). It appears as CS genes act as a double-edged sword whose loss or gain of expression leads to premature aging or cancer respectively.

The comparison between XP and CS unveils an intriguing scenario with respect to cancer induction. XP patients are 1,000 times more prone to developing cancer while CS patients, in contrast, do not develop it (Zhang et al., 2016). Interestingly, loss of CS proteins in cancer prone INK4a/ARF−/− mice protected them from skin cancer development illustrating a negative correlation between expression of CS proteins and carcinogenesis (Lu et al., 2001). Observations of increased expression of CS proteins in cancer cells and reduction of neoplastic growth by suppression of CS proteins suggest that CS proteins are intimately associated with carcinogenic processes. In corroboration, induction of pro-survival pathways by CS proteins leading to cell robustness and increased proliferation has been recently demonstrated (Caputo et al., 2013; Paccosi et al., 2021; Filippi et al., 2022). Unlike CS patients, humans afflicted with Werner, Bloom and Rothmund-Thomson syndromes display increased cancer incidence. Strikingly, increased expression of RecQ helicases and RecQL4 in particular is observed in many human cancer types. In sharp contract to CS, loss of RecQ helicases is associated with cancer development processes. Nevertheless, increased expression of CS and RecQ helicase proteins appears to be a common phenomenon in cancer cells. Given the uniqueness of CS genes in both premature aging and cancer either by loss or increased expression, CS can be an ideal model system for dissecting the molecular pathways involved in premature aging and carcinogenesis.

## The unbalance of CS proteins in aging and cancer

*CSA* and *CSB* genes were initially characterized as the main players of TCR, wherein CSA and CSB proteins first participate in the removal of the RNA polymerase stalled ahead of the lesion (Bregman et al., 1996; Svejstrup, 2003) and then in the recruitment of NER proteins, including the transcription/DNA repair factor TFIIH (Lainé and Egly, 2006; van der Weegen 2020). It has become increasingly clear that some of the features exhibited by CS patients could hardly be attributed to TCR deficiency alone and that CSA and CSB functions extend their roles far beyond of DNA repair. Indeed, studies over the last decades demonstrated that CS proteins participate in other cellular processes: (I) basal and activated transcription as well as in the recovery of RNA synthesis after the massive transcriptional shut down induced upon genotoxic stresses (Balajee et al., 1997; Proietti-De-Santis et al., 2006; Kristensen et al., 2013; Epanchintsev et al., 2017; Lee et al., 2019; Epanchintsev et al., 2020) (II) Modulation of p53 levels in response to different cellular stresses to re-equilibrate the physiological response in favor of cell survival and proliferation instead of cell cycle arrest and cell death (Latini et al., 2011); (III) Maintenance of mitochondrial homeostasis (Aamann et al., 2010; Berquist and Wilson, 2012; Chatre et al., 2015) (IV) Regulation of autophagy and lysosomal function (Scheibye-Knudsen et al., 2012; Majora et al., 2018) and transcription of RNA polymerase I for ribosomal biogenesis (Alupei et al., 2018; Okur et al., 2020; Lanzafame et al., 2021) and (V) Regulation of cell division completion through triggering the abscission of the intercellular bridge at the end of cytokinesis (Paccosi et al., 2020). Dysregulated expression of CS proteins has been shown to exhibit differential health outcomes: loss of function by mutations in CS genes invariably leads to complex premature aging phenotypes, and elevated expression of CS proteins is associated with carcinogenesis. The lack of cancer incidence observed in CS patients (Zhang et al., 2016) and CSB knockout mouse model system (Lu et al., 2001) is in agreement with the increased apoptotic potential reported in CS cells upon exposure to genotoxic agents. The probability of developing cancer causing mutations is expected to be extremely low in CS cells, that display an elevated apoptotic potential, as it was demonstrated in a study performed on human CSB and hamster UV61 (carrying a mutation in the homolog of the human *csb* gene) cells: indeed, both cell lines displayed an increased apoptotic response following UV exposure compared with normal cells, thus avoiding the onset of mutations (Balajee et al., 2000). This phenomenon is presumably related to the cytotoxic effect of lesions in the coding regions of the genome, that determine the stalling of RNA pol II and lead to transcription arrest and subsequent stimulation of apoptotic response. Along this line, another study reported that the UV-induced mutation frequency in CS cells is lower than in normal cells suggesting that TCR deficiency may be protective against UV-induced mutagenesis (Reid-Bayliss et al., 2016) by stimulating a robust apoptotic response.

Although the precise role(s) of CS genes in carcinogenesis is not clearly elucidated, increased expression of CS genes confers proliferative and survival advantage to cancer cells. It is likely that cancer cells activate the pro-survival and antiapoptotic pathways by modulating the expression of CS genes. Nevertheless, the contrasting phenotypes of premature aging and carcinogenesis mediated by CS gene products present an ideal model system for developing new therapeutic strategies for aging and cancer.

### CS proteins as a biological predictor for cell fate determination

The tumor suppressor p53 protein participates in multiple cellular response pathways that protect the cells from the deleterious effects of many stress-inducing agents inclusive of DNA damage, oncogenic activation, hypoxia, and other forms of stress (Hong et al., 2014). Cellular DNA damage response is aimed either to cause a transient or a permanent cell cycle arrest, leading to either cell death via apoptosis or cellular senescence (Feng et al., 2008; Bieging et al., 2014) or, alternatively, to prevent damaged cells from undergoing neoplastic transformation as p53 is the most frequently mutated gene in human cancers (Guimaraes and Hainaut, 2002).

A complex network of feedback loop mechanisms controls the action of this protein (Tyner et al., 2002; Brown et al., 2009). Indeed, an altered (reduced or increased) p53 activity would be detrimental either by resulting in cancer or premature aging through increased proliferative advantage or replicative senescence, respectively (Rodier et al., 2007).

**FIGURE 2:**
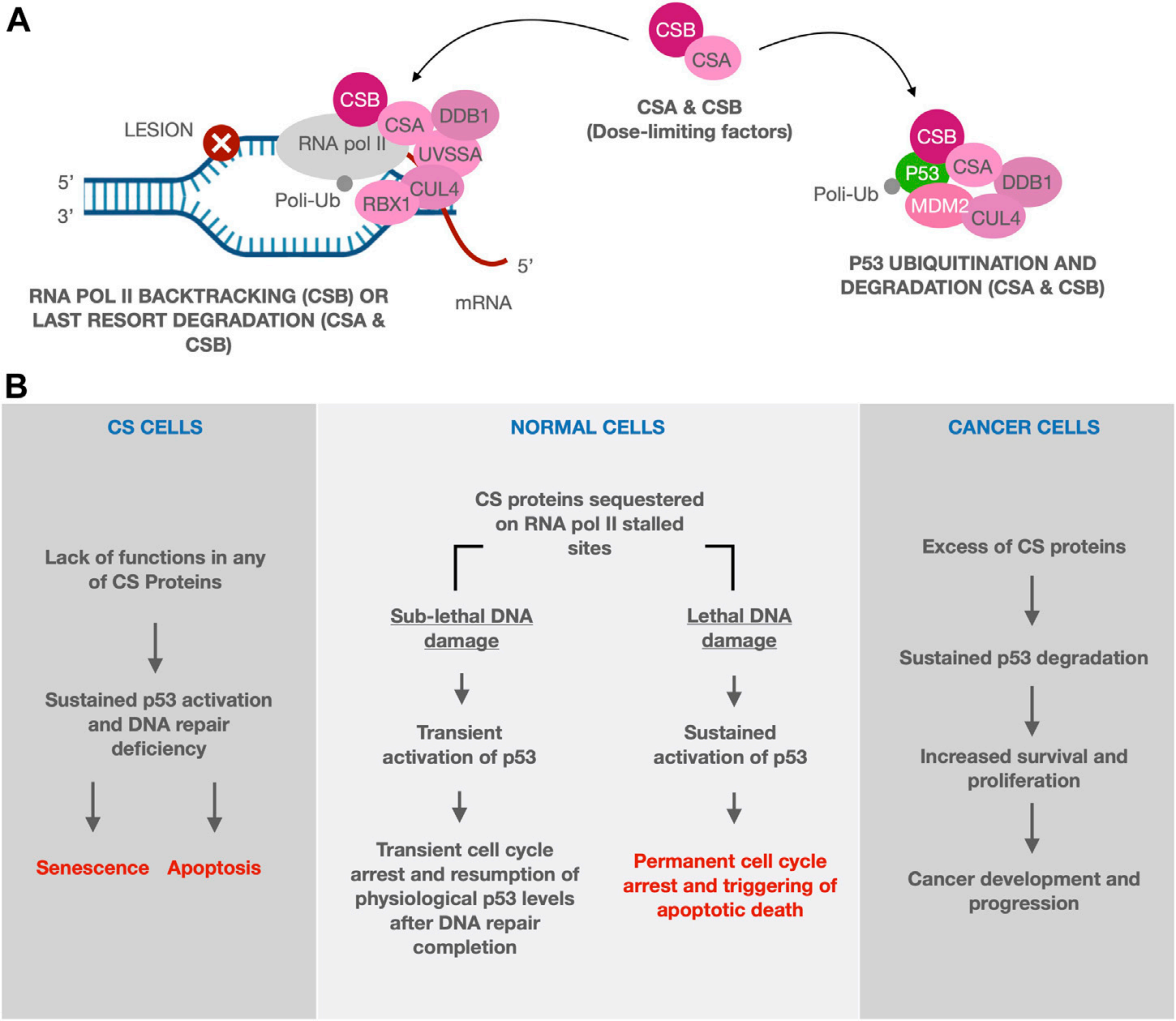
The biological predictor role of CS proteins. In our model CS proteins, which are alternatively involved in DNA repair or p53 ubiquitination/degradation, act as dose-limiting factors. When a cytotoxic lesion occurs, CS proteins, together with the TCR machinery, are transiently recruited at the level of the stalled RNA pol II, whose backtracking or degradation will allow the repair in case of sub-lethal damage. In this context, p53 is not ubiquitinated and degraded and it is free to induce a transient cell cycle arrest, in those cells receiving sub-lethal DNA damage. Cell cycle will be then restored upon the completion of DNA repair when CS proteins, being no longer engaged in TCR, will be able to re-establish the basal level of p53 through its ubiquitination and degradation. If cells are exposed to lethal damage/stress, instead, they will undergo an irreversible cell cycle arrest followed by apoptosis, due to the sustained activation of p53. In this case, CS proteins accumulate and persist at the damaged sites and, as a result of this entrapment, p53 may not be efficiently degraded [**(A,B)** central panel]. When CS role as biological predictors is missing, as in the context of CS cells, the lack of p53 degradation leads to a massive induction of apoptosis [**(B)** left panel]. Instead in cancer cells, where CS proteins are overexpressed, abnormally high levels of p53 degradation are induced, thereby promoting development and progression of cancers, by shifting the cell fate toward survival and proliferation rather than apoptosis [**(B)** right panel].

Given the recently unveiled role of CS proteins in counteracting p53 activity by indirect/direct stimulation of its degradation (Berquist and Bohr, 2011; Latini et al., 2011; Frontini and Proietti-De-Santis, 2012), it was recently proposed that CSA and CSB, besides their authentic roles in DNA repair, may also act as dose-limiting factors for p53 activity (Paccosi and Proietti-De-Santis, 2021). In brief, CS proteins are able to drive p53 to the MDM2-dependent ubiquitination/degradation, therefore down regulating the cellular levels of p53. Moreover, expression of CSB is induced by p53 itself, as a part of a feedback loop (Latini et al., 2011). This mechanism allows the cells to resume physiological levels of p53 after the transient up-regulation of the p53 response pathway induced by genotoxic agents and aimed to temporarily arrest the cell cycle and potentiate the DNA repair mechanisms. When a cytotoxic lesion occurs, CSB is recruited to push the backtracked RNA pol II forward. Alternatively, the entire TCR complex will be recruited for the repair and the eventual last resort degradation of RNA pol II, both processes being mediated by CSA and CSB (Wilson et al., 2013; Kokic et al., 2021). Indeed, high levels of unrepaired DNA damage would sequester CS proteins at the damaged sites in which RNA pol II is stalled and, as a result of the limited availability of CS proteins, p53 may not be efficiently degraded (Fousteri et al., 2006; Frontini and Proietti-De-Santis, 2012). The resulting sustained p53 activity will lead to cell death. The foregoing account suggests that CS proteins, through modulation of p53 activity, have a crucial role in determining cellular fate between survival and apoptosis. In this regard, CS proteins act as biological predictors of cell fate after sub-lethal and lethal DNA damage induction (Figures 2A,B, central panel).

An imbalance in the equilibrium between p53 and CS proteins might be responsible for either the accelerated aging process observed in CS patients or the promotion of cancer in tissues that overexpress CS proteins. In the context of CS cells, the lack of p53 degradation leads to a massive induction of apoptosis that may account for the overall loss of tissue homeostasis found in CS patients (Rapin et al., 2006) (Figure 2B, left panel). On the other hand, overexpression of CS proteins likely induce abnormally high levels of p53 degradation, thereby promoting development and progression of cancers, by shifting the cell fate toward survival and proliferation rather than apoptosis (Figure 2B, right panel).

### Loss of CS proteins and premature aging

Cockayne Syndrome recapitulates many molecular traits of physiological aging, such as DNA repair dysfunction, oxidative DNA accumulation, impaired redox balance, mitochondrial dysfunction, chromatin remodeling defects, and transcription deregulation (Karikkineth et al., 2017; Pascucci et al., 2018), each of these defects being reasonably relevant in contributing to the clinical phenotypes of CS patients (Cleaver et al., 2013; Lanzafame et al., 2013; Scheibye-Knudsen et al., 2013; Vélez-Cruz and Egly, 2013). CS cells are characterized by a stronger apoptotic response to DNA damaging agents than normal cells (Balajee et al., 2000; Liu et al., 2006; Laposa et al., 2007) that can account for the progressive loss of organ homeostasis and lack of tissue renewal observed in CS patients (Rapin et al., 2006). Regarding DNA repair dysfunction, CS cells display a defective repair mechanism for cyclobutane dimers (Barrett et al., 1991; Parris and Kraemer, 1993), and DNA single strand breaks (Spivak and Hanawalt, 2006). Oxidative DNA lesions have been reported in both human and mouse tissues (Brooks, 2007; Kirkali et al., 2009; Wang P. et al., 2012) and *in vitro* experiments demonstrated that CS cells are defective in repairing oxidized bases unlike normal cells (Spivak and Hanawalt, 2006; D’Errico et al., 2017). It was previously noticed that CSA or CSB deficient primary fibroblasts fail to degrade RNA pol II after UV irradiation, leading to the hypothesis that a deficiency in RNA pol II processing and prolonged transcription arrest in response to DNA damage, rather than a compromised TCR activity, may underlie the CS-like neurodegenerative phenotype (Proietti-De-Santis et al., 2006; Nakazawa et al., 2012).

The recent evidence for the involvement of CSA and CSB proteins in the repair of mitochondrial DNA damage both upon oxidative stress and in the electron transport chain (Aamann et al., 2010; Kamenisch et al., 2010; Pascucci et al., 2012; Sykora et al., 2012; Scheibye-Knudsen et al., 2013) led to the hypothesis that mutations in CSA and CSB may influence the redox homeostasis and mediate the cellular hypersensitivity to oxidative agents. In corroboration, an altered redox balance has been reported in primary fibroblasts derived from CSA and CSB patients, with changes in cellular bioenergetics, alterations in oxidative metabolism, glycolysis and osmoregulation (Pascucci et al., 2012). Additionally, a higher rate of mtDNA mutations was observed both in human cells from CS patients and aged *Csa* and *Csb* mutant mice (Kamenisch et al., 2010). Other studies performed on *CSB* ^*m/m*^ mouse cells revealed a highly abnormal and increased mitochondrial content, due to reduced autophagy and an increased free radical production (Scheibye-Knudsen et al., 2012). Since the failure of autophagy has been linked to neurodegeneration (Komatsu et al., 2006), a role was suggested for CS proteins in counteracting mitochondrial mutagenesis that can minimize the neurodegenerative and aging processes which are typical of CS (Kamenisch et al., 2010; Karikkineth et al., 2017). Available data suggest that both mitochondrial leakage of byproducts of oxidative phosphorylation (Barnes and Lindahl, 2004; Wallace, 2005) and mitochondrial autophagy (Koren and Kimchi, 2012) can lead to neurodegeneration and aging. In fact, specific abnormalities related to mitochondrial dysfunction have been observed in the Purkinje cells of *CSB* ^*m/m*^ mice (Laposa et al., 2007). Even if mitochondrial dysfunction may account at least for some of the neurodegenerative features observed in CS patients, some cytological abnormalities found in the brains of CS patients, such as the appearance of binucleated neurons and multinucleated astrocytes (Itoh et al., 1999; Weidenheim et al., 2009), appear to arise from a defect in cell division due to the lack of CSA or CSB, which have recently been shown to exert a role in the last step of cytokinesis, the abscission (Paccosi et al., 2020).

The notion that ribosomal impairment may be a driver for the aging processes dates back to the 1960s, when it was proposed that errors in the translation process would be worsened if accompanied by ribosomal proteins impairment (Orgel, 1963), and it took the name of “error catastrophe theory of aging” (Gallant et al., 1997). Inspired by this original hypothesis, a novel pathomechanism has been proposed recently in CS cells, in which an abnormal RNA polymerase I (RNA pol I) transcription activity was shown to affect ribosomal performance, inducing both misfolded proteins (Alupei et al., 2018; Qiang et al., 2021) and nucleolar stress, with the latter characterized by a p53-regulated cell cycle arrest and senescence and/or apoptosis. The decreased RNA pol I transcription is followed by ribosomal malfunction, loss of proteostasis, and Endoplasmic reticulum (ER) stress-induced inhibition of rRNA synthesis all of which lead to death of CS cells. This kind of pathomechanism might explain both developmental defects and neurological degeneration observed in CS (Phan et al., 2019).

Given the complex clinical phenotypes of CS, it is difficult to explain each of the phenotypic traits based on a specific defect such as TCR. CS may be a multifactorial disease where most of the features are initiated primarily by the loss of CS genes and secondarily by the impairment of CS associated signaling pathways.

### CS proteins overexpression in cancer

Recent studies have demonstrated that a number of cancer cell lines of different tissue origin display a dramatic up-regulation of CS proteins expression and are dependent on increased levels of CS proteins for their survival. Indeed, upon suppression of CSA or CSB proteins in these cells, several pro-apoptotic factors become dramatically up-regulated leading to a massive induction of apoptosis. Strikingly, ablation of CS proteins specifically affects the tumor cells, without any impact on non-transformed cells, suggesting that the increased expression of CS proteins is crucial for cancer cell survival (Caputo et al., 2013; Paccosi et al., 2021; Filippi et al., 2022).

How CS proteins participate in cancer development and progression? First of all, CSB has been proven to act as a mediator of the hypoxic response by redistributing the transcriptional co-activator p300 between hypoxia-inducible factor 1 (HIF1) and p53 (Filippi et al., 2008; Frontini and Proietti-De-Santis, 2009). Hypoxia is a prevalent feature of solid tumors and cancer cells have to deal with micro-environmental stress (Schito et al., 2006) by developing tolerance to hypoxia by increasing the vascularization that can support their growth (Harris, 2002; Gorgoulis et al., 2018).

A regulatory network of proteins is required either for p53-induced cell death or for hypoxic adaptation. This may occur at the gene level, and involves transcriptional induction through binding of these respective factors (p53 or HIF-1) to responsive elements at the promoter of the downstream genes (Amelio and Melino, 2015). In this context, CSB plays a role in adaption to hypoxia by activating and downregulating the HIF-1 and p53 transcriptional programs, respectively (Filippi et al., 2008). While in normal healthy cells hypoxic stress activates the p53 response that leads to the expression of genes involved in cell death, instead, in tumor cells, HIF-1 plays a central role in adaptation to hypoxia by activating genes implicated in angiogenesis, such as *vegf* and *gapdh*, that favor the conditions for cancer progression (Harris, 2002; Gorgoulis et al., 2018). Hence, in the absence of functional CSB, cells are unable to react to hypoxic stimuli and to activate transcription of crucial pro-survival genes. Therefore, suppression of CSB activity might reduce the hypoxia tolerance of tumor cells, thus increasing their apoptotic threshold (Proietti-De-Santis et al., 2018).

Cancer cells also require increased expression of CS proteins for dealing with other kind of stresses, such as oxidative and ER stresses, the latter responsible of the triggering of the Unfolded Protein Response (UPR), the adaptive survival strategy that cancer cells adopt to deal with the increasing levels of ER stress (Storz et al., 2005; Yadav et al., 2014). Indeed, it was demonstrated that CSB is involved in regulating the levels of misfolded proteins by maintaining a productive ER protein folding environment through up-regulation of mediators of UPR pro-survival pathway. Also, it was shown that CSB suppression leads to both up-regulation of pro-apoptotic factors downstream of the ATF3-CHOP cascade, which are responsible for the massive induction of apoptosis, and down-regulation of the UPR pro-survival mediators (Caputo et al., 2017). This observation suggests that ablation of CSB in cancer cells results in an increase in pre-existing ER stress that tilts the balance from pro-survival towards apoptosis (Wang et al., 2014).

It is well documented that CS proteins participate in RNA pol I and II mediated basal and activated transcription (Balajee et al., 1997; Selby and Sancar, 1997; Bradsher et al., 2002; Yuan et al., 2007; Brooks et al., 2008; Koch et al., 2014; Lanzafame et al., 2021). In this context, it is worth noting the role of CSB in the transcriptional activation of some key genes, as *neuroD1* (Ciaffardini et al., 2014), whose tight regulation is well known to be required to avoid the trigger of dysregulatory mechanisms for initiating and promoting oncogenic activities (Costanzo et al., 2022). For this reason, it might be of interest investigating if CSB overexpression is causative for the abnormal regulation of these key genes and, consequently, for the induction of the hyperactivation cascades aimed to direct cell fate towards abnormal survival and proliferation.

Although most studies regarding the CS involvement in cancer has been focused on CSB overexpression, a similar role for CSA can not be excluded. Filippi and collaborators provided the first evidence that Breast Cancer (BC) cells displayed an increased expression of CSA protein, and that its ablation by AntiSense Oligonucleotides (ASOs) drastically impaired the tumorigenicity of BC cells by hampering their survival and proliferative capabilities without affecting normal breast cells. Moreover, CSA ablation also resulted in lowering the IC_50_ value of Oxaliplatin and Paclitaxel, two commonly used chemotherapeutic agents in breast cancer treatment (Filippi et al., 2022).

Furthermore, unpublished data from our laboratory indicate that protein kinase B (AKT) is subjected to a CSA-dependent ubiquitination that regulates its membrane recruitment and, consequently, its phosphorylation/activation, thus tuning the activation the PI3K-AKT pathway, which is known to be over-activated in many human cancers (Revathidevi and Munirajan, 2019; Uko et al., 2020). These observations led us to hypothesize that also CSA overexpression may favor cancer development and progression by activating the pro-survival AKT pathway and that both CSA and CSB have the potential to serve as new molecular therapeutic targets for cancer.

**FIGURE 3:**
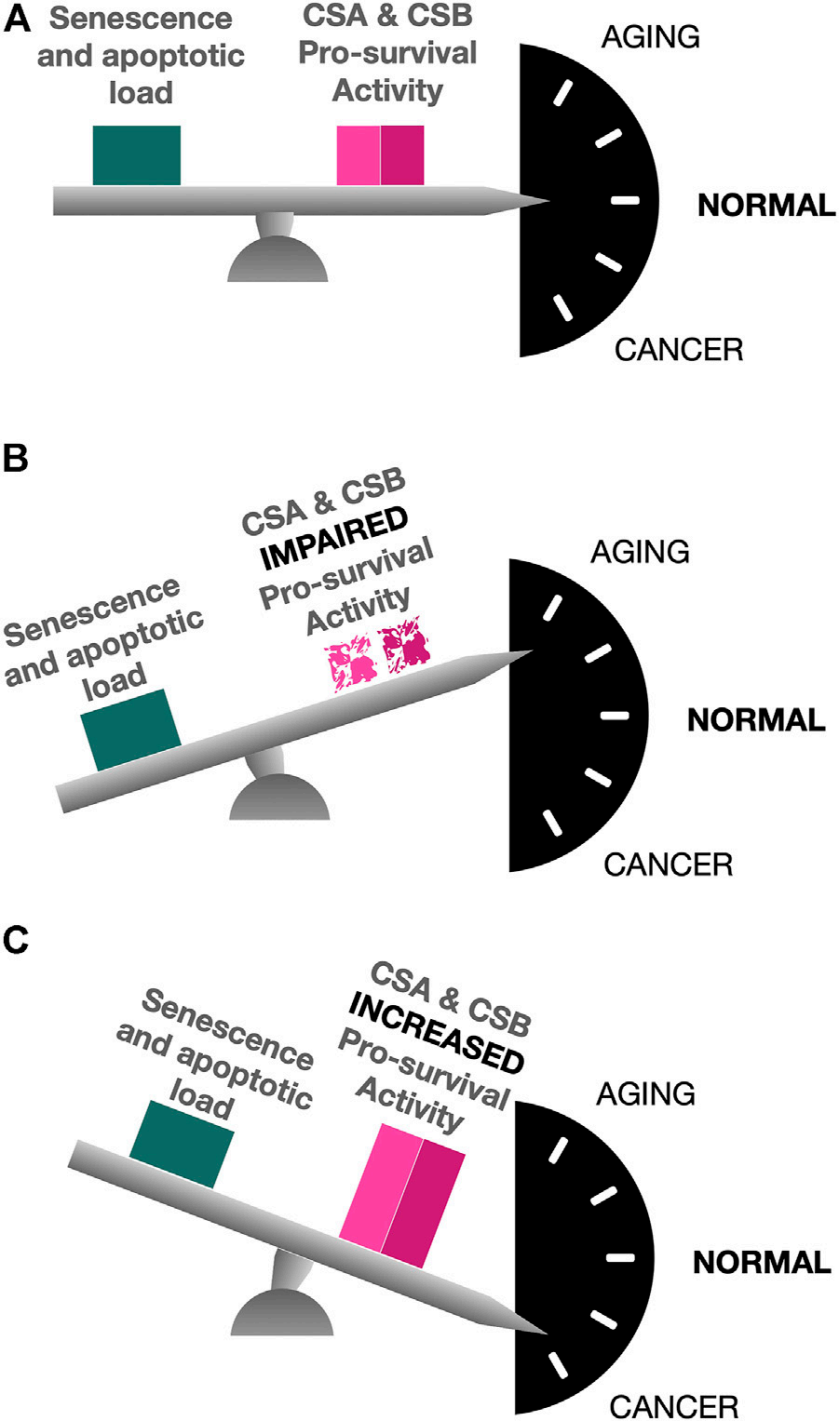
The balanced action of CS proteins. In normal conditions, CSA and CSB pro-survival activity counterbalances the signaling pathways that, otherwise, would lead to the induction of senescence and the triggering of apoptosis in response to the insults to which the cells are daily exposed **(A)**. Lack of CS proteins due to gene mutations causes an unbalance in which senescence and apoptotic load are not counteracted, thus leading to complex premature aging phenotypes **(B)**. A gain of CS proteins expression, instead, is responsible for an up-regulation of pro-survival pathways, thus favoring conditions for cancer development and progression **(C)**.

In conclusion, when the delicate balance of CS proteins expression is disrupted, cells are forced to face a plethora of complex molecular alterations directed either to favor cancer development and progression, in case of CS gain of expression, or to establish a process of accelerated aging, in case of CS proteins loss of expression (Figure 3).

## Future perspectives and strategies for intervention

Mutations in DNA repair genes give rise to progeroid diseases where different DNA metabolic activities are deregulated/disrupted (Kamenisch and Berneburg, 2009). Progeroid syndromes are usually characterized by an increased and earlier incidence of cancer, that confirms the role of genomic instability as a promoting factor for both aging and cancer processes (Schumacher et al., 2008; Wolters and Schumacher, 2013) and this was also demonstrated in old mice, in which decline of stem-cell function with age has been correlated to DNA damage, which leads to dramatic epigenetic changes and alterations in gene expression, damage and instability with increased probabilities for malignant transformation (Rossi et al., 2007). In sharp contrast to RecQ helicase deficient premature aging syndromes characterized by increased cancer susceptibility, CS patients are not associated with cancer development showing even a sort of resistance to cancer development (Lu et al., 2001). These two distinct outcomes from the loss and gain of expression of CS proteins in premature aging and cancer warrant future investigations for using the CS proteins as novel therapeutic targets for aging and cancer.

### Strategies for CS patients care and their potential use for counteracting normal aging

Given the importance of endogenous ROS production in the pathogenesis of CS, an ideal approach is to administer antioxidants to reduce the oxidative stress in CS patients. A similar approach in the mouse model of yet another human neurodegenerative disorder Ataxia Telangiectasia Mutated (ATM) increased the lifespan by reducing the neurological symptoms (Gueven et al., 2006). It remains to be investigated whether the antioxidant approach will be effective for therapeutic intervention of CS patients because this kind of treatment may trigger some unfavorable side effects, such as DNA damage and induction of apoptosis (Fox et al., 2012), with the risk of enhancing the loss of cells/tissues and evetually tissue homeostasis. Another example of focused approach is the lithium chloride or rapamycin treatment, that was to work well in reversing the mitochondrial phenotype of *CSB* ^*m/m*^ cells by increasing autophagy (Scheibye-Knudsen et al., 2012). This kind of treatment yet to be tested for other neurodegenerative diseases treatment (Wang et al., 2012).

The ribosomal biogenesis and protein synthesis in CS cells may be normalized by treatments with pharmaceutical chaperones, such as 4-phenylbutyrate (4-PBA) and Tauroursodeoxycholic acid (TUDCA), both of them being able to reduce ER stress. Interestingly, these chaperones were shown to block both the protein synthesis and the hypersensitivity of CS cells to oxidative stress (Alupei et al., 2018). Moreover, treatment with pharmaceutical chaperones was shown to restore cellular growth, impaired transcription initiation by RNA pol I and protein synthesis, opening a promising scenario for treating those clinical symptoms related to impaired ribosome functioning (Qiang et al., 2021). Interestingly, the emerging knowledge of a common, unifying molecular mechanism underlying the pleiotropic action of CSA and CSB proteins in the cascade of events leading to ubiquitin/proteasome-directed protein degradation in a plethora of different processes (Ratner et al., 1998; Proietti-De-Santis et al., 2006; Latini et al., 2011; Epanchintsev et al., 2017; Okur et al., 2020; Paccosi et al., 2020) may not only reasonably explain the plethora of cellular functions that are impaired when either CSA or CSB gene is mutated, but also open a new and intriguing scenario for the study of the molecular basis of CS. In this context, it has been recently proposed that the identification of the ubiquitin-proteasome machinery, able to comprehensively face the different molecular aspects of CS, as a new potential therapeutic target for intervention could open a promising avenue to design effective therapeutic interventions, whether confirmed and corroborated by *in vivo* studies (Paccosi and Proietti-De-Santis, 2021). Despite treating each of the CS defects separately, restoration of *csa* and *csb* gene expression would be the best strategy for intervention. In this regard, the feasibility of retinal gene therapy for CS yet to be proven in a murine model (Gruntman et al., 2015). Moreover, CRISPR/Cas9-mediated gene correction has shown to be successful in rescuing CS induced pluripotent stem cells from premature aging defects, laying a foundation for the development of novel therapeutic strategies to treat the overall CS symptomatology (Wang et al., 2022). Last but not least, in the context of a wide heterogeneity of clinical features and severity of symptoms among the CS patients (Natale, 2011), recent molecular technologies involving next generation sequencing s may play pivotal role in investigating the etiology of the disease. Calmels and colleagues performed an interesting work on a large cohort of patients with Cockayne syndrome, and demonstrated that the human mutation spectrum of the CS genes is not yet saturated and that there is a plethora of genetic variants still to be identified, since a definitive correlation between genotype and phenotype is still missing (Calmels et al., 2018). Recently, many novel *csb* variants associated with severe or mild clinical phenotypes (Friedman et al., 2021; Yousefipour and Mahjoobi, 2021; Zayoud et al., 2021; Duong et al., 2022) have been identified by whole-genome and/or whole-exome sequencing. CS being a disease with no effective treatments or cure, the kind of molecular approach described above will open up new avenues for facilitating diagnosis and will likely improve the definition of the sketchy genotype-phenotype relationship in patients with CS. The emerging knowledge of the molecular mechanisms underlying CS raises hope not only that some of these strategies will be successful in improving life and health for CS patients, but also that these kind of approaches may be a useful tool against some deleterious features related to the normal aging, such as DNA damage sensitivity, loss of proteostasis and neurodegeneration.

### CSA and CSB targeting for cancer treatment

In addiction to the observation of the dramatic up-regulation of CS proteins in a number of cancer cell lines of different tissue origin (Caputo et al., 2013; Paccosi et al., 2021; Filippi et al., 2022), it is worth nothing that several groups also provided evidences that many *csb* single-nucleotide polymorphism (SNPs) are associated with increased cancer susceptibility or affected response to chemotherapy: i) the rs2228526, rs4253160, rs12571445, and rs3793784 SNPs of *csb* may contribute to the susceptibility of lung cancer (Lin et al., 2008; Ma et al., 2009); ii) the SNP rs4253002 has shown a significant association with gastrointestinal toxicity in the patients receiving Platinum-Paclitaxel (TP) regimen, while the SNP rs4253212 has shown to be correlated with neutropenia in the patients receiving Platinum-Gemcitabine (GP) regimen, suggesting that CSB might be involved in regulating clinical outcomes of platinum-based chemotherapy (Song et al., 2017). These data suggest that, almost regarding *csb* gene, both overexpression and SNPs may contribute to cancer development and progression. However, these findings need to be validated by larger studies with diverse populations and also functional evaluations. Regarding *csa* gene, instead, there is still a sketchy knowledge regarding both the association between SNPs/cancer predisposition and the mechanisms by which CSA overexpression may contribute to cancer development. The established data are that CS proteins play a major role in cancer progression and that their ablation by antisense technology not only results in increased levels of apoptotic death of cancer cells but, most importantly, does not affect the normal cells, suggesting that the former are addicted to high levels of CS proteins, an ideal condition for any candidate therapeutic approach. Moreover, the sensitivity of tumor cells either to the chemotherapeutic agent Cisplatin or to Oxaliplatin and Paclitaxel, was shown to be increased after silencing *csb* or *csa* genes respectively by RNA interference (Proietti De Santis et al., 2018; Filippi et al., 2022). This is a key point for minimizing the chemotherapeutic dose required to induce apoptosis, thereby reducing chemotherapy side effects. Worthy of note, it has been also demonstrated that CSA ablation even restores drug sensitivity in oxaliplatin-resistant cells (Filippi et al., 2022). In conclusion, the challenging task is to understand whether CS proteins may be an attractive candidate for therapeutic targeting. To achieve this milestone, further studies, both *in vitro* and *in vivo,* are mandatory in order to fully elucidate the contribution of CSB and CSA in cancer development and progression, in the optic of paving the way for a new kind therapeutic approach which, to date, seems to be really attractive.
